# The Immune-Centric Revolution Translated into Clinical Application: Peripheral Blood Mononuclear Cell (PBMNC) Therapy in Diabetic Patients with No-Option Critical Limb-Threatening Ischemia (NO-CLTI)—Rationale and Meta-Analysis of Observational Studies

**DOI:** 10.3390/jcm13237230

**Published:** 2024-11-28

**Authors:** Laura Rehak, Laura Giurato, Matteo Monami, Marco Meloni, Alessia Scatena, Andrea Panunzi, Giada Maria Manti, Carlo Maria Ferdinando Caravaggi, Luigi Uccioli

**Affiliations:** 1 Athena Cell Therapy Technologies, 50126 Florence, Italy; laurarehak@gmail.com (L.R.); giada.manti@athena-med.it (G.M.M.); 2 Department of Biomedicine and Prevention, Diabetes-Endocrine Section CTO Hospital, Tor Vergata University of Rome, 00133 Rome, Italy; lauragiurato@yahoo.it (L.G.); andreapanunzi@gmail.com (A.P.); 3 Department of Diabetology Azienda Ospedaliera Universitaria Careggi, University of Florence, 50134 Florence, Italy; matteo.monami@unifi.it; 4 Diabetic Foot Unit, Department of Systems Medicine, Tor Vergata University of Rome, 00133 Rome, Italy; meloni.marco@libero.it; 5 Diabetology Unit, San Donato Hospital Arezzo, Local Health Authorities Southeast Tuscany, 52100 Arezzo, Italy; alessia.scatena@uslsudest.toscana.it; 6 PhD School of Applied Medical and Surgical Sciences, University of Rome Tor Vergata Italy, 00133 Rome, Italy; 7 Diabetic Foot Unit, IRCCS MultiMedica Milan, 20099 Milan, Italy; caravaggi@me.com

**Keywords:** peripheral blood mononuclear cell, PBMNC, cell therapy, no-option CLTI, immune-centric revolution, diabetic foot, wound healing

## Abstract

Chronic limb-threatening ischemia (CLTI), the most advanced form of peripheral arterial disease (PAD), is the comorbidity primarily responsible for major lower-limb amputations, particularly for diabetic patients. Autologous cell therapy has been the focus of efforts over the past 20 years to create non-interventional therapeutic options for no-option CLTI to improve limb perfusion and wound healing. Among the different available techniques, peripheral blood mononuclear cells (PBMNC) appear to be the most promising autologous cell therapy due to physio-pathological considerations and clinical evidence, which will be discussed in this review. A meta-analysis of six clinical studies, including 256 diabetic patients treated with naive, fresh PBMNC produced via a selective filtration point-of-care device, was conducted. PBMNC was associated with a mean yearly amputation rate of 15.7%, a mean healing rate of 62%, and a time to healing of 208.6 ± 136.5 days. Moreover, an increase in TcPO2 and a reduction in pain were observed. All-cause mortality, with a mean rate of 22.2% and a yearly mortality rate of 18.8%, was reported. No serious adverse events were reported. Finally, some practical and financial considerations are provided, which point to the therapy’s recommendation as the first line of treatment for this particular and crucial patient group.

## 1. Introduction

Diabetic foot is a syndrome that includes long-term complications of diabetes and comorbidities. It is responsible for major lower-limb amputations, the loss of quality of life, and a reduction in lifespan. Chronic limb-threatening ischemia (CLTI) is the comorbidity primarily accountable for major lower-limb amputations [1,2,3,4].

Despite the relevant progress recorded in the treatment of CLTI in diabetic patients over the last 20 years and the availability of endovascular techniques that allow the revascularization of very distal arteries, there are still patients for whom limb salvage procedures are not satisfying [5].

The most frequent reasons are very poor lower-limb vascular arterial trees with unsolvable technical difficulties that hamper any therapeutic approach and/or relapse after apparent successful revascularization [6,7].

Diabetic patients with below-the-ankle (BTA) arterial disease are the peripheral arterial disease (PAD) patients with the worst outcomes and the highest amputation rate [8]. Sometimes, revascularization procedures are impossible because of the complete absence of any visible BTA artery or the impossibility of overcoming stenosis or obstructions in these small vessels, often presenting calcific plaques. This condition is called “desert foot” [6,7].

Those patients have a very high risk of major lower-limb amputations, and in addition, because of the significant presence of comorbidities such as coronary artery disease and heart failure, they face the highest risk of premature death [9].

Recently, regenerative medicine through autologous cell therapy has offered new opportunities to treat this specific group of patients, defined as diabetic patients with no option CLTI (NO-CLTI). This definition relates to the previous failure of any revascularization procedure, the persistence of ischemia, and a non-healing ulcer [8] with few, if any, chances of healing [8,10,11,12,13]. Different sources of cells, such as bone marrow mononuclear cells (BMM-MNCs), adipose-derived stem cells (ADSCs), or nano-fragmented adipose tissue, have been utilized to perform cell therapy in patients with different success rates [14,15,16,17,18].

Among the different techniques available, autologous cell therapy (ACT) with peripheral blood mononuclear cells (PBMNCs) appears to be the most promising due to physio-pathological considerations and clinical evidence [19,20,21,22,23,24,25,26]. The aim of this review is to analyze the physio-pathological rationale behind the PBMNC autologous cell therapy to treat diabetic patients with CLTI, as presented in Part I. We also carefully analyzed the clinical evidence through a meta-analysis of PBMNC studies on CLTI in the second part of the review. Finally, we share some economic and practical considerations that suggest this therapy as the first choice for this specific and critical group of patients.

**Part I—rationale of PBMNC therapy in diabetic patients with no-option critical limb ischemia.**

Extensive research has delved into the potential mechanisms underlying DFUs, with previous studies primarily concentrating on diabetic peripheral vascular disease, neuropathy, and wound infections [27,28,29]. In diabetic wounds, tissue ischemia, hypoxia, a high glucose microenvironment, and chronic inflammation disrupt the natural progression of healing stages, leading to either delayed healing or non-healing of the wounds and various clinical complications [30,31,32]. PAD is one of the most critical factors in the appearance and healing processes of foot ulcers: nearly 70% of diabetic patients referred to a specialized diabetic foot unit presented an ischemic diabetic foot ulcer [5]. Approximately a quarter of patients with symptomatic PAD experience BTA distribution disease [6,7]. BTA distribution disease and/or small artery disease (SAD) affect up to 25% of patients with symptomatic PAD [33]. Poor clinical outcomes regarding wound healing, time to healing, limb salvage, and survival are related to such disease patterns [33,34,35,36,37,38,39].

Researchers have increasingly explored the roles of immune cells, endothelial cells, keratinocytes, and fibroblasts in wound healing, suggesting that modulating molecular signaling pathways, whether upregulated or downregulated, play a crucial role in DFU healing [14,25].

PBMNCs are involved in both angiogenesis and the immunoregulation ability in DFU healing, as observed in patients with no-option CLTI [14,23,25].

PBMNCs are composed mainly of hematopoietic lineage cells, including most lymphoid cells and myeloid monocytes, and less of stem/progenitor cells, such as hematopoietic stem/progenitor cells (HSPCs and EPCs) [40].

PBMNCs exert their effect through a paracrine mechanism, releasing cytokines, chemokines, growth factors, lipids and extracellular vehicles (EVs), and mRNA [41,42,43,44]. These biological cascades induced via PBMNCs promote angiogenesis and a permissive local environment for adequate cell replacement and the restoration of tissue integrity, also through the considerable modulation of the regenerative activity of resident stem/progenitor cells [25,45,46,47].

PBMNC autologous cell therapy has the ability to work simultaneously on two different mechanisms of action: angiogenesis-inducing collateral vessel formation and, in addition, the immuno-modulatory effect through the polarization of inflammatory macrophages of the M1 phenotype into regenerative M2 macrophages.

PBMNCs promote angiogenesis.

PBMNCs can positively regulate the formation of new blood vessels through the complex tie between the secretion of proangiogenic mediators and the modulation of endothelial cell (EC) and endothelial progenitor cell (EPC) activities (e.g., homing) [48,49]. As a result, angiogenesis and tissue healing are accelerated when immune cell behavior is managed in a timely manner [40].

It is well known that pro-arteriogenic monocytes/macrophages expressing receptor TIE2 promote neovascularization in the ischemic limb and are upregulated in patients with CLTI [48,50,51,52]. Preclinical studies have demonstrated extensively that the secretomes of PBMNCs exhibit significant tissue-regenerative and proangiogenic abilities [42,43,44,53,54].

PBMNCs’ angiogenic capacity is physiologically triggered via hypoxia; this, in turn, induces the expression of monocyte chemoattract protein 1 (MCP-1), which is required for the secondary recruitment of monocytes and the maximal stimulation of angiogenesis [49,55,56,57,58]. Moreover, recent data challenge the long-held belief that HIF1α-mediated metabolic remodeling can be induced cell-autonomously through hypoxia alone [59]. Optimum HIF1α activation in the wound-edge epithelium requires IL-17A, provided via lymphocyte T-cells [59].

Vagesjo et al. demonstrated, for the first time, that macrophages are not only critical cells in angiogenesis and arteriogenesis but also regulate the blood flow in ischemic muscle [58]. These data align with the angiogenesis and arteriogenic ability of monocytes/macrophages reported in previous studies [60,61,62,63,64,65,66]. Gurevich et al. [66] demonstrated, through the live imaging of wound angiogenesis, that macrophages orchestrated vessel sprouting and regression, suggesting a pivotal role of macrophages in tissue healing [66,67]. Lymphocytes also play a synergic role in the angiogenic mechanism. Regulatory T-cells (Tregs) contribute to angiogenesis in ischemic tissue via apelin-mediated sprouting in diabetic patients [68,69,70,71,72]. Accordingly, the absence of lymphocytes hampers macrophage polarization and angiogenesis during diabetic wound healing [31]. Additionally, CD8+ T-cells appear to regulate vascular regeneration [73].

**Figure 1:**
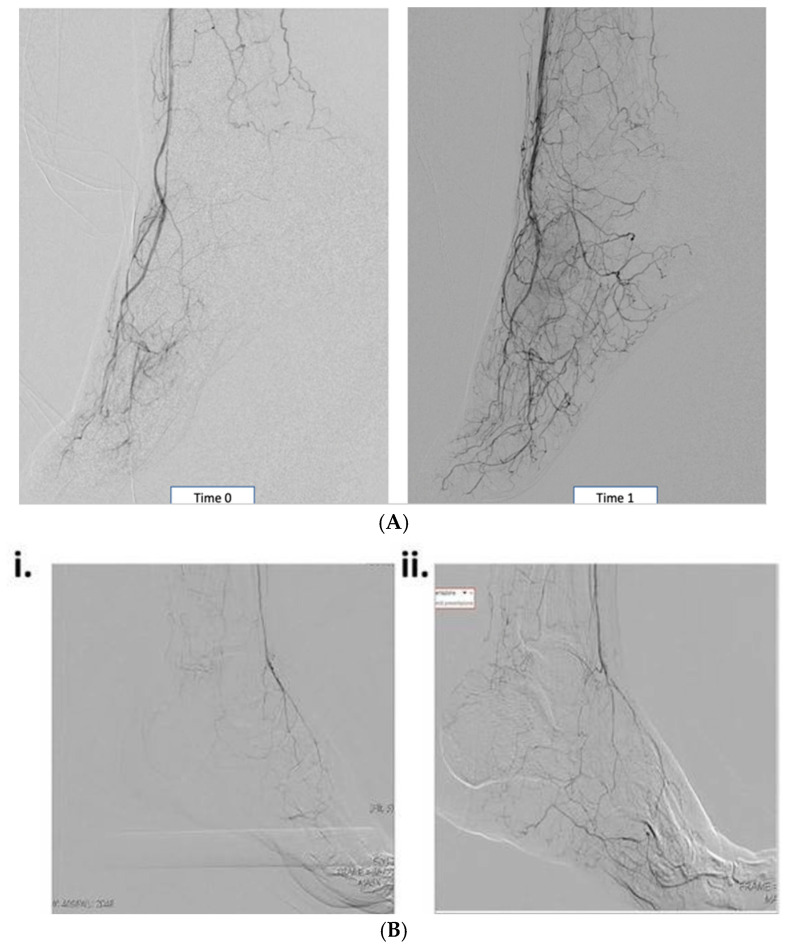
Angiography pre- and post-PBMNC implants. (**A**) Pre- (Time 0) and post-14 weeks (Time 1) after a PBMNC implant in diabetic patients with no-option CLTI: the sprouting of new vasa interspersed through all of the foot (unpublished data from the University of Tor Vergata, Rome). (**B**) (i) Patient after unsuccessful PTA (TcPO2 < 30 mmHg) therapy showing the typical “desert” foot condition; (ii) patient after 2 months of PB-MNC therapy showing collateral vascular remodeling (Panunzi et al. [20]).

PBMNCs, produced via a point-of-care selective filtration system with an indication for use for human cell therapy from 100 to 120 mL of peripheral blood, maintain a high angiogenic capacity in vitro and in vivo [74]. In vivo, in an animal model of CLI, a histological analysis of ischemic tissues demonstrated that PBMNC implants were associated with a significant increase in regenerated capillary and arteriole formation [74,75]. Accordingly, the angiography of diabetic patients with CLTI before and after implants with PBMNCs produced through selective filtration showed new collateral formation and remodeling [20,22] (Figure 1A,B).

Interestingly, naive autologous PBMNC implants, produced with the same selective filtration device, led to angiogenesis in a diabetic patient affected by untreatable vasculogenic erectile dysfunction [76].

Angiogenesis and collateral remodeling were also confirmed via TcPO2 values increasing after PBMNC implants [20,24]. TcPO2-increase data from naive PBMNC produced after selective filtration is comparable to those shown by Dubsky et al. [77], which were revealed after the implant of mobilized PBMNCs produced via apheresis. Panunzi et al. [20] observed that patients healed after PBMNC implants without a major amputation, showing significantly higher TcPO2 levels (47.9 ± 22.1) compared to patients without ulcer healing (34.6 ± 20.5) (*p* = 0.0395) at one year. The authors also observed that TcPO2 values remained stable in both groups at two years of follow-up (49.2 ± 20.2 vs. 39.3 ± 22.8, *p* = 0.1265). Interestingly, the authors confirmed that TcPO2 improvement was more significant in cases where PBMNC implants were repeated three times. Furthermore, no significant correlation was seen between CD34+ and CD34 + CXCR4+ frequency and TcPO2, demonstrating that angiogenic potency was unrelated to stem cell CD34+ concentrations, in keeping with previously reported results [78,79,80]. Vice versa, Moriya et al. [78] previously correlated the positive clinical outcome effect in terms of limb salvage, wound healing, and pain reduction with VEGF plasma concentrations after a PBMNC implant in ischemic muscle. Notably, Panunzi et al. also observed that patients with high tissue perfusion (TcPo2 > 40 mmHg) correlated with a higher number of small-size EVs released from PBMNC implants, suggesting that angiogenesis signals are correlated to EVs’ release from PBMNCs [20].

Finally, it has been demonstrated that the administration of exogenous macrophages successfully improved angiogenesis and skeletal muscle regeneration, suggesting that macrophage-based cell therapy may be a promising adjunctive therapeutic approach to peripheral arterial disease and CLTI [81,82,83,84,85,86,87].

PBMNC immuno-modulatory effect (M1 to M2 macrophages’ polarization).

Resolving ischemia through revascularization (PTA or bypass) is not always sufficient to induce wound healing, especially in diabetic patients [33,88,89,90].

Chronic wounds are characterized by persistent inflammatory conditions of the macrophage inflammatory M1 phenotype, the most represented group of cells [30,31,32,91,92,93]. In diabetic patients, M1 macrophages drive the elevated and prolonged non-resolving inflammatory phase that characterizes non-healing ulcers. Pro-inflammatory M1 macrophages make up about 80% of the cells at the chronic wound margin, and substantial evidence from studies on both humans and animals suggests that the transition to M2-like regenerative phenotypes may not happen as planned, given that macrophage polarization is essential for the wound healing process to advance [94,95,96]. Interestingly, diabetic wounds not only initially exhibit an excess of M1 macrophages but later display a deficiency in M2 macrophages during the proliferative stages, indicating that changes in macrophage activation may hinder diabetic wound healing [97,98,99]. The persistent inflammation and increased numbers of M1 macrophages in diabetic wounds lead to the reduced proliferation and migration of keratinocytes, fibroblasts, and endothelial cells necessary for wound healing [95].

Wood et al. [100] observed a significant delay in macrophage infiltration in diabetic wounds due to reduced CCL2, also known as monocyte chemoattractant protein 1 (MCP-1), expression. Accordingly, MCP-1 promotes healing in diabetic foot wounds by restoring the correct macrophage response, suggesting that restoring the correct kinetics of the macrophage response can be an attractive strategy for promoting healing [100].

Therefore, correcting this abnormal macrophage behavior in chronic wounds could enhance wound healing [25,75,98,101,102,103,104,105]. Increasing the M2 macrophage concentration in wounds leads to an increased release of anti-inflammatory cytokines, reducing inflammation and increasing the growth factors necessary for proliferation, migration, and the repair process [25,28,106,107,108]. Interestingly, a retrospective trial on 367 CLTI diabetic patients showed that, despite successful revascularization, the wound healing of lesions on the foot was extremely slow, requiring up to 2 years [109]; this finding suggested that oxygen perfusion alone cannot induce tissue regeneration and that other biological mechanisms, such as increased M1 levels and a reduced M2 polarization switch, are involved. On this basis, Persiani et al. [24] suggested, for the first time, that PBMNC cell therapy can represent an adjuvant treatment in treating CLTI diabetic patients after revascularization.

The implanting of PBMNCs in chronic wounds and diabetic foot promotes macrophage polarization from the inflammatory phenotype M1 to the regenerative phenotype M2 [25,45,46,47,110].

**Figure 2:**
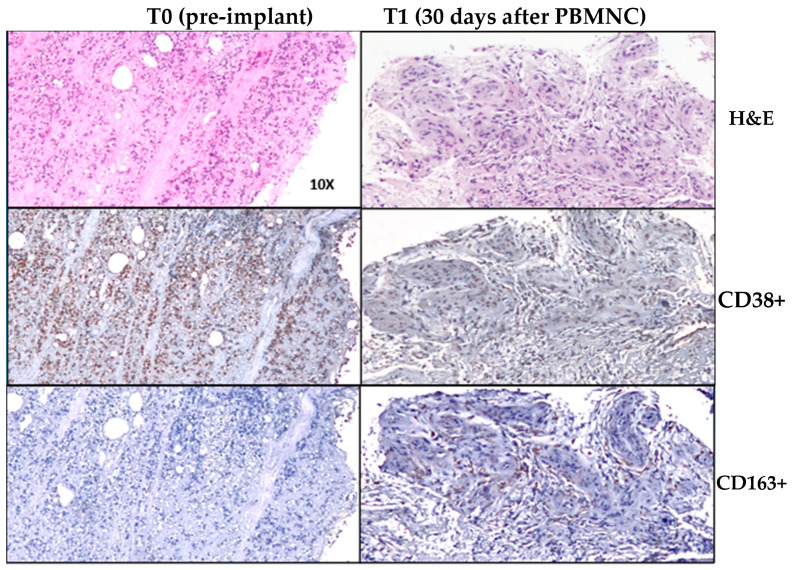
Morphological aspects and an immunohistochemical analysis from biopsies taken pre- (T0) and 30 days post-PBMNC implant (T1) in a diabetic foot wound area (M1 (CD38+) and M2 (CD163+)). At the top (first row), hematoxylin and eosin (H&E) staining shows an inflamed diabetic wound due to ulceration at Time 0 and an important area of regenerated tissue at Time 1. In the second row, at T0, CD38+ immunostaining highlights a large M1 macrophage inflammatory infiltration (10×), and at T1, the quantity and intensity of CD38+ staining for M1 were both reduced (10×). In the third row, at T0, rare M2 macrophages were positive for the CD163 marker (10×), whereas the amount of CD163+ M2 cells significantly increased at Time 1 (10×). Courtesy of Manuela Montanaro (Department Biomedicine and Prevention) and Alessandro Mauriello (Department Experimental Medicine-University of Tor Vergata Roma) (Unpublished data Policlinico Tor Vergata Roma). Immunohistochemistry and immunofluorescence methods are described in the Materials and Methods section.

**Figure 3:**
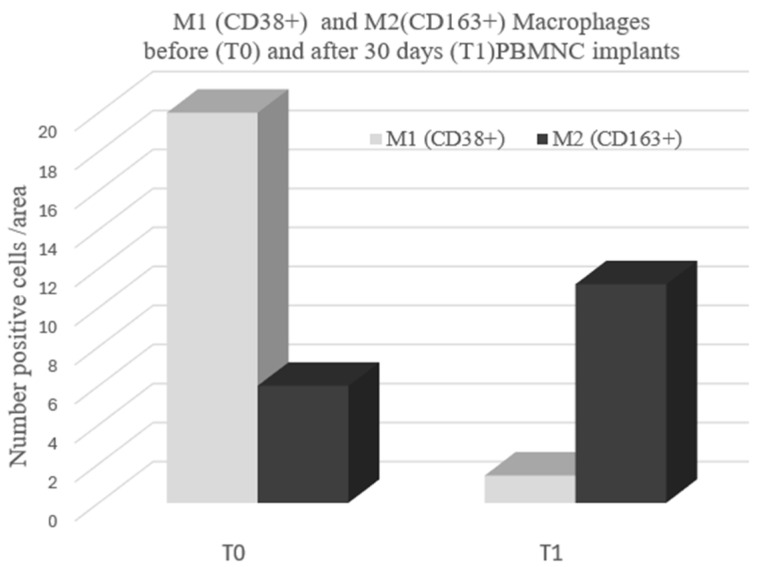
Macrophage polarization quantification expressed in the number of positive cell/area (CD38+ M1 and CD163+ M2) measured in immunohistochemical samples from a wound area before (T0) and after 30 days (T1) a PBMNC implant in three representative patients (*n* = 3). Courtesy of Manuela Montanaro (Department Biomedicine and Prevention) and Alessandro Mauriello (Department Experimental Medicine- University of Tor Vergata Roma); unpublished data, Policlinico Tor Vergata Roma. Immunohistochemistry and immunofluorescence methods are described in the Materials and Methods section.

**Figure 4:**
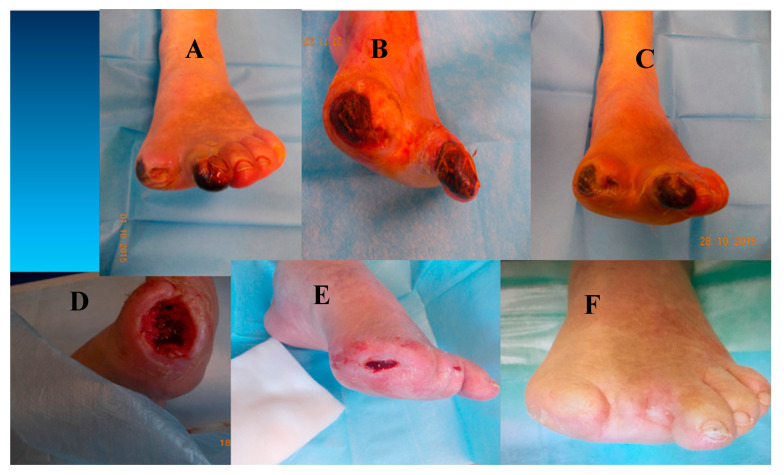
Clinical case of diabetic patient with no-option CLTI before and after three implants of PBMNCs. Clinical pictures: from the upper left at the time of the first PBMNC implant to the lower right six months later. Pictures were taken at the baseline (**A**), after the first, second, and third PBMNC implants (**B**–**D**), one month after the final PBMNC implant (**E**), and at a 6-month follow-up (**F**).

Interestingly, studies have demonstrated the same ability in different tissues and pathologies [65,110,111,112,113,114,115]. PBMNC produced through a selective filtration implant has been shown to induce both angiogenesis and polarization from M1 to M2 in a diabetic foot ischemic lesion, inducing healing [75]. Moreover, using a previously described method [116], unpublished data from DFU of Policlinico Tor Vergata Roma confirmed the ability of PBMNCs produced via selective filtration to polarized M1 to M2 through an immunohistochemical analysis from biopsies of the wound foot area of a diabetic patient with NO-CLTI (Figure 2 and Figure 3). Figure 4 shows the clinical evolution of the same patient at the baseline and after each PBMNC implant until complete healing was achieved six months later.

So far, according to the current knowledge, PBMNC is the only autologous cell therapy in which both angiogenic and M2 polarization effects have been demonstrated in diabetic CLTI patients. This complex mechanism of action could at least partially explain the metanalyses data of Rigato et al. [17], which showed that only PBMNCs, but not other autologous cell therapies such as bone marrow mononuclear cells (BMMNCs) or bone marrow mesenchymal cells BM-MSCs, were effective in significantly reducing amputations and increasing the amputation survival (AFS) of patients with NO-CLTI.

Moreover, macrophage polarization is a critical step in pain control, and PBMNC implants showed the ability to reduce pain [117,118].

Macrophages exert a significant influence over pain modulation: transitioning into the M2 phenotype can inhibit inflammation, promote tissue healing, and relieve neuropathic pain [119,120]. Recent research has increasingly spotlighted the role of M2 macrophages in reducing pain, suggesting that manipulating these M2 macrophages can effectively mitigate pain [118]. M2 macrophages secrete substances like IL-10, opioids, and specialized pro-resolving mediators (SPMs), which interact with relevant receptors on nociceptive sensory neurons [118,121,122,123]; M2 macrophages transmit their miRNAs and mitochondria to nociceptive sensory neurons via extracellular vehicles (EVs) [124,125].

**Part II—Metanalysis of clinical studies: PBMNC therapy in diabetic patients with no-option critical limb ischemia.**

To validate the clinical effect of PBMNC implants in diabetic patients with no-option CLTI, we conducted this systematic review and meta-analysis in conformity with the PRISMA checklist [126] and following a previously published protocol [127]. The search strategy (Figure S1—study flow summary) and selection criteria (Table S1—detailed information on the search string strategy) are reported in the Supplementary Material.

## 2. Materials and Methods

This meta-analysis is part of a wider meta-analysis of studies on diabetic foot syndrome [127]. The present analysis includes all studies (both controlled and noncontrolled), including diabetic patients with foot ulcers, PBMNCs, and no-option-CLI, for a duration of at least 26 weeks. Animal studies were excluded, whereas no language or date restriction was imposed. A Medline and Embase search was performed up to February 1st, 2024, using the following search string: (autologous or “stem cells” or “stem cell” or “cell therapy” or “cellular therapy”) and “ulcer” and “diabetes”. Therapy with autologous cells obtained from bone marrow or adipose tissue was excluded. Expanded, manipulated, or allogenic cell concentrates were excluded. Table S1 (Supplementary Material) reports detailed information on the search strategy. Further studies were manually searched for references from retrieved papers.

Selection criteria:

To be eligible, a study had to have enrolled patients with CLTI, diabetes, and foot ulcers. Only studies obtaining PBMNCs from peripheral blood cells not mobilized via granulocyte colony-stimulating factor were included. All patients showed a TcPO < 30 mmHg.

Two independent reviewers (L.U. and M.M. Careggi) screened all the titles and abstracts of the identified studies for inclusion. Discrepancies were resolved by a third, independent reviewer (M. M. Tor Vergata).

Data extraction and collection:

The variables of interest were major and minor amputation, all-cause mortality, ulcer healing, time to healing, pain, TcPO2, ABI, quality of life, and peri-procedural complications, as previously reported [126].

Data extraction was performed independently by two of the authors (L.U. and M.M. Careggi), and conflicts were resolved by a third investigator (M.M. Tor Vergata).

Titles and abstracts were screened independently by the authors, and potentially relevant articles were retrieved as full text. For all published trials, the results reported in published papers and supplements were used as the primary source of information. When the required information on protocols or outcomes was not available in the main or secondary publications, an attempt at retrieval was performed by consulting the clinicaltrials.gov website.

Endpoints:

The primary endpoint was major amputation. The secondary endpoints were minor amputation, all-cause mortality, ulcer healing, time to healing, pain, transcutaneous oxygen pressure (TcPO2), and serious peri-procedural adverse events.

Statistical analyses:

Heterogeneity was assessed through an I2 test, whereas funnel plots were used to detect publication bias for principal endpoints with at least 10 trials.

If data from more than one study on a given outcome were available, a meta-analysis using a random-effects model as the primary analysis was performed. Mantel–Haenzel odds ratios and 95% confidence intervals (MH-OR and 95% CIs) were either calculated or extracted directly from the publications. Weighted mean differences (WMDs) and 95% CIs were calculated for continuous variables.

All analyses were performed using Review Manager (RevMan), Version 5.3 (Copenhagen: The Nordic Cochrane Centre, The Cochrane Collaboration, 2014), for RCT and Comprehensive Meta Analysis, v. 2.

Immunohistochemistry and immunofluorescence:

The immunohistochemistry data reported in Figure 3 and Figure 4 were obtained using a previously described method [116]. In brief terms, in order to evaluate the different states of macrophage activation and polarization in treated and untreated groups, a semiquantitative evaluation of CD68 (KP1 mouse monoclonal antibody, general macrophage marker, 1:200; Abcam, Cambridge, UK), CD38 (rabbit monoclonal, typically M1 macrophage marker, 1:100; Abcam), and CD163 (rabbit monoclonal, typically M2 macrophage marker, 1:500; Abcam) expressions was performed.

To carry out immunohistochemistry reactions, antigen retrieval was performed on 3 µm-thick paraffin sections using a Tris-EDTA (ethylenediaminetetraacetic acid) citrate buffer, pH 7.8, for 30 min at 95 °C. Sections were then incubated for 1 h at room temperature with primary antibodies. Washing was performed with PBS (phosphate-buffered saline)/Tween20, pH 7.6. HRP-DAB Novolink Detection Kit (Leica Biosystem, Nussloch, Germany) revealed reactions. All markers were evaluated with the support of digital software (ImageViewer, Ventana, Roche, Basel, Switzerland) by 2 blind observers who counted the number of positive cells; the results were reported as the number of positive cells/mm2. To confirm immunohistochemical results and analyze the co-localization of CD68 and CD163 markers, a confocal microscopy analysis was carried out. Briefly, 3 to 4 µm-thick paraffin sections were dewaxed and dehydrated; then, antigen retrieval was performed using a Tris-EDTA citrate, pH 7.8, buffer for 10 min in a microwave stove.

Auto-fluorescence was reduced via a tetrahydroborate solution for 40 min. Sections were then incubated overnight at 4 °C with a CD68 antibody (1:200) diluted in 5% goat serum. Washing was performed with PBS/Tween (0.1%), and the sections were incubated for 1 h at room temperature with the CD163 antibody (1:1000) diluted in 5% goat serum. After the washes, the slides were incubated for 1 h with the appropriate secondary antibodies conjugated using Alexa Fluor 488 or 568 (Thermo Fisher, Waltham, MA, USA) and DAPI (1 µg/mL). The slides were then mounted using ProLong Antifade (Thermo Fisher). Images were acquired with a Nikon A1 confocal laser microscope (Nikon, Tokyo, Japan).

## 3. Results

The study flow summary is reported in Figure S1 of the Supplementary Material. Six studies fulfilling all inclusion criteria were retrieved. Four studies were cohort prospective studies [20,21,22,24], and two were nested case-control studies [19,26]. All studies produced naive autologous PBMNC through a point-of-care system based on selective filtration starting from 120 mL of peripheral blood to obtain a mean of 200 million total nuclear cells (TNCs) and 100 million PBMNCs in 10 mL of physiological solution [74]. The mean age, proportion of women, and baseline HbA1c were 72.1 years, 33.2%, and 7.5% (57 mmol/mol), respectively. All studies used intramuscular transplantation as a route of administration.

The main characteristics of the included studies enrolling 256 patients are reported in Table 1.

### 3.1. Major Amputation

**Figure 5:**
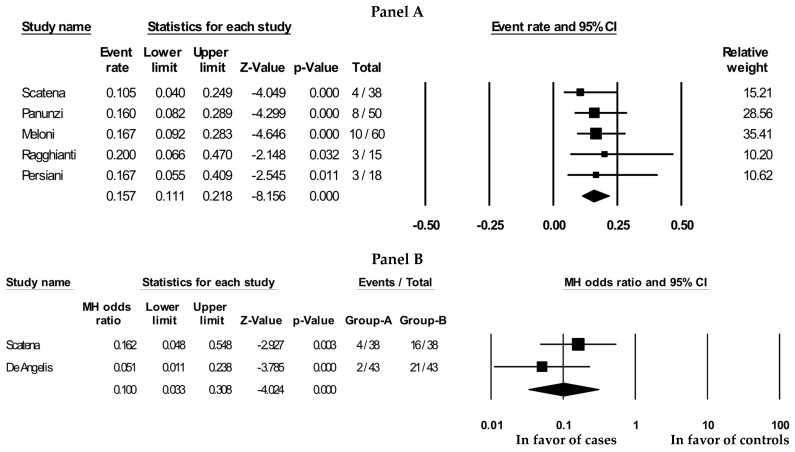
Major amputation with PBMNCs in all included studies (**A**), yearly rate and in case-control studies (**B**), at the endpoint. Reported references are Scatena et al [19], Panunzi et al, [20], Ragghianti et al [21], Meloni et al [22], Persiani et al [24], De Angelis et al [26].

All the included studies reported this outcome at 1 year, except for two studies [19,20] with FU at 2 years. PBMNCs were associated with a mean yearly amputation rate of 15.7% (event rate: 15.7 [11.1, 21.8], *p* < 0.001; I2 = 0%; Figure 5A).

Out of six studies, only two [19,26] were designed as nested case-control studies showing a significant reduction in major amputation in favor of PBMNCs at the endpoint (2 years; MH-OR: 0.10 (0.03;0.31), *p* < 0.001; I2: 25%; Figure 5B).

For Scatena et al. [19], the study group comprised 76 patients: 38 in the standard-care control group and 38 in the PBMNC group. No significant difference in age, gender, diabetic status (type, disease duration, and glycated hemoglobin), lesion site, or several comorbidities between the two groups was recorded. At the same time, the retinopathy prevalence rate was higher in the standard therapy group. The PBMNCs group showed a significant improvement in all recorded primary outcomes. The Kaplan–Meier curves and the log-rank test results showed a significantly lower amputation rate in the PBMNC group (*p* = 0.000; Figure 2) at each follow-up point (1, 3, 6, 12, and 24 months). Only four amputations in 38 patients (10.5%) were observed in the PBMNC group, while the control group included 15 amputations (39.5%) (*p* = 0.0037).

De Angelis et al. [26], in a prospective and not randomized study, compared a PBMNC group of patients who did not respond to conventional therapy (*n* = 43) versus a historically matched control group. Patients of both groups were suffering from CLI Fontaine scale IV with chronic ulcers and comorbidities. The PBMNC-treated group showed a statistically significant improvement in limb salvage of 95.3% versus 52.2% for the control group (*p* < 0.001), and the result was maintained for two years.

### 3.2. Figures on Ulcer Healing and Time to Healing

Out of six studies, four [19,20,21,22] reported this outcome: PBMNCs were associated with a mean healing rate of 62% (event rate: 62 [43,79], *p* = 0,22; I2 = 821; Figure 2). Information on the time to healing was reported in only two studies [19,20], with a mean of 208.6 ± 136.5 days, *p* = 0.12; I2: 97%.

**Figure 6:**
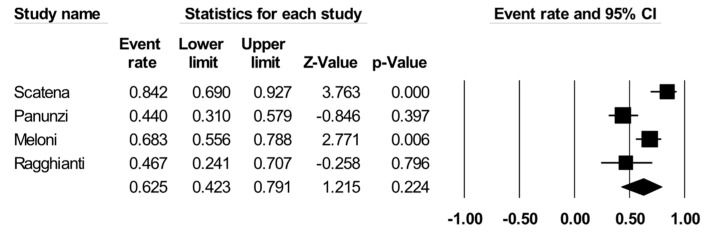
Ulcer healing rate with PBMNCs in all included studies. Reported references are Scatena et al [19], Panunzi et al, [20], Ragghianti et al [21], Meloni et al [22].

One study comparing PBMNCs with the standard of care [19] reported a significant reduction in favor of PBMNCs (MH-OR: 197 [23;1727], *p* < 0.001). Ulcer healing is represented in Figure 6.

### 3.3. All-Cause Mortality

**Figure 7:**
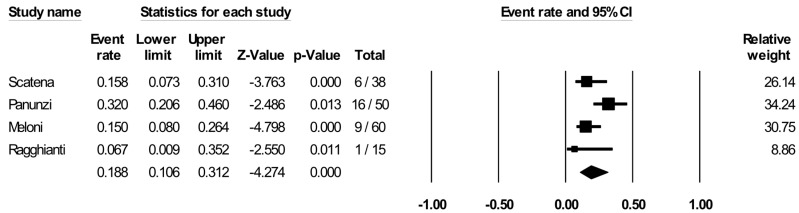
All-cause mortality (yearly rate) with PBMNCs in all included studies. Reported references are Scatena et al [19], Panunzi et al, [20], Ragghianti et al [21], Meloni et al [22], Persiani et al [24], De Angelis et al [26].

All included studies, except two [24,26], reported information on all-cause mortality, with a mean rate of 22.2% (event rate: 22.2 (9.4, 44.0), *p* = 0.015; I2 = 90%). The number of deaths was 42 with a yearly mortality rate of 18.8% (Figure 7). One study comparing PBMNCs with the standard of care [19] reported a significant reduction in favor of PBMNCs (MH-OR: 0.07 (0.02, 0.21), *p* < 0.001).

### 3.4. Other Outcomes: TcPO2 and Pain

Five [19,20,21,22,24] and four [19,21,22,24] studies reported information on TcPO2 and pain (measured with a 10-point visual analog scale) at the endpoint, respectively. No information was reported for ABI. Data on TcPO2 vs. standard care were not available in any study. The baseline average values for TcPO2 were 14.3 mmHg (measured before a PBMNC implant). The mean increase in TcPO2 in comparison with the baseline values after a PBMNC implant was 22.7 mmHg (WMD: 22.7 (20.8;24.6), *p* < 0.001; I2:42%). Data on TcPO2 vs. the standard care were not available in any study.

The baseline values for pain were 6.8/10. Pain was reported as an endpoint value (no data on differences between the endpoint and the baseline were available) with a mean value of 1.8 points (WMD: 1.8(0.2;3.3), *p* = 0.023; I2:85%). The percentage mean reduction in reported pain between the baseline and the endpoint was 73.5%.

### 3.5. Safety: Adverse Events

All trials reported information on peri-procedural serious adverse events (SAEs). Any SAEs were reported.

### 3.6. PBMNC Concentrations and Implants

All the papers considered in this review used the same point-of-care medical device and protocol to concentrate PBMNCs.

The concentration of autologous PBMNCs was produced using a Pall Celeris/Hematrate Blood filtration system from Cook Regentec/MonoCells Athena Cell Therapies Technologies, a filtration-based point-of-care device for the rapid preparation of total nuclear cells (TNCs) from 100 to 120 mL of anticoagulated blood for use in human cell-therapy applications.

This system is a point-of-care device developed to concentrate an MNC-enriched population of TNCs with high angiogenic potential from peripheral blood without apheresis or density gradient/centrifugation, employing a selective filtration system. The cell product obtained was extensively characterized by composition, recovery, and FACS cell population analysis [74].

In brief terms, TNCs were enriched 2.97-fold, and MNCs were enriched 4.2-fold (average dose of PBMNCs implanted = 1.06 +/− 0.28 × 10^8^); the CD34+ progenitor cell subpopulation was enriched by 5.6%–4.2% versus peripheral blood with a mean CD34+ cell count of 1.37 × 10^6^. The efficiency of the CD34+ hematopoietic stem cell enrichment of this selective filtration system is comparable with the CD34+ concentration obtained using a point-of-care device for bone marrow cells (BMAC2) [74]. In each treated patient was an implanted PBMNC dose of 1 × 10^8^.

In all the studies, PBMNC concentrations and implants were performed in an operating room with anesthesiologic support (propofol and/or peripheral block) after the appropriate surgical debridement of the wound bed or after the third implant and increased TcPO2.

PBMNCs were implanted through multiple perilesional and intramuscular injections of 10 mL PBMNC cell suspensions (0.2–0.3 mL in boluses) and BTA following the pathway of the wound-related artery at intervals of 1–2 cm and to a mean depth of 1.5–2 cm, using a 21 G or 25 G needle in relation to the site of administration. This procedure was repeated two or three times for each patient at intervals of 30–45 days from each other in each study.

## 4. Discussion

For the first time in this paper, we present a systematic review and meta-analysis on naive autologous PBMNC, delving into the physio-pathological rationale behind this cell therapy and the clinical evidence that supports its use through a meta-analysis of six clinical observational studies. This novel research aims to provide a fresh perspective on the treatment of patients with no-option CLTI.

Regarding the physio-pathological rationale of PBMNC for treating patients with no-option CLTI, in the past, trials have historically focused on stem cell populations. At the same time, recently, several studies have highlighted the influence of local immune responses on stem cell function [25,45,46,128,129]. Monocytes/macrophages and lymphocytes have emerged as key players in tissue repair processes [25,129,130,131,132,133]. The paradigm shift from stem cell-based to immune cell-based therapy is now well known as the immune-centric revolution [25,45,46,128,134,135,136]. A seminal study published in *Nature* further supported this paradigm shift by demonstrating that stem cell therapy did not primarily improve heart function by generating new cardiomyocytes but, rather, by activating macrophages and inducing an immune response [134,135]. The study suggests that the therapeutic benefit is more likely linked to the local and acute immune response, rather than the intrinsic regenerative capacity of stem cells themselves, underscoring the pivotal role of immune modulation in tissue-repair mechanisms [134,135].

Additionally, increasing data suggest that diabetes significantly compromises stem cell populations both in bone marrow [14,17,137,138,139,140,141] and in adipose tissue [142,143,144,145,146]. Adipose-derived stem cells (ADSCs) isolated from the ischemic limbs of diabetic patients showed a reduced angiogenic potency compared to control-group nondiabetic ischemic patients [147]. Interestingly, a recent study demonstrated the ability of endothelial cell secretomes to reverse the harmful effects of high glucose concentrations on ADSCs in order to enhance their ability to participate in angiogenesis and wound healing [148]. In 2010, for the first time, Cramer et al. [149] compared ADSCs derived from non-diabetic and diabetic donors regarding glucose metabolism, cell replication, apoptosis, and differentiation potential, concluding that an elevation in glucose reduces the proliferative capacity of diabetic ADSCs significantly. Overall, these results indicate that the impaired function of mesenchymal stem cells that reside in adipose tissue, as one of the significant sources of adult stem cells, might be responsible for complications related to type 2 diabetes [149]. Interestingly, results from Navarro et al. in 2014 suggest that adipose tissue-resident monocytes (SVF CD14+ cells) are the critical population able to contribute to tissue vascularization because they are more efficient in inducing angiogenesis than ADSCs contained in the stromal vascular fraction. Moreover, the same study, through a quantitative analysis of angiogenesis at 14 days after cell implants, demonstrated that neovascularization due to adipose tissue-resident monocytes SVF CD14+ cells or peripheral blood monocytes was 2 or 3.5 times higher than angiogenesis observed in ASC implants [150]. A few years after, in 2018, Cai et al. [151] observed accordingly that the adipose tissue depletion of macrophages resulted in incompetent angiogenesis, reduced stem cell recruitment, and also even a poor retention rate, while upregulated macrophages allowed for better angiogenesis and survival, suggesting that macrophages release secreted angiogenic factors and influenced blood-derived stem cell infiltration crucial for tissue revascularization. Accordingly, in the following years, more recent papers confirmed that inflammation strongly reduces the regenerative ability of mesenchymal stem cells [127,144,152,153]. This observation may at least partially explain the negative results reported in randomized trials of diabetic patients with no-option CLTI treated with bone marrow stem cell-based therapy in terms of major amputation and the AFS of bone marrow stem cell-based therapy [154,155,156,157,158,159]. Rigato et al. [17] confirmed these data, which showed, in a separate analysis, that cell therapy with PB-MNCs, but not BM-MNCs or BM-MSCs, was associated with a significant improvement in amputation and amputation-free survival. CD34+ hematopoietic stem cells are considered partially compromised in diabetic patients. Instead, PB-MNC seems to be less affected [69,160,161].

In line with this rationale, our meta-analysis conducted on observational studies of naive PB-MNC showed a robust angiogenic and wound healing effect, with a comparable clinical outcome for these highly critical diabetic patients [19,20,21,22,24,26]. Despite the lack of randomized studies on naive PBMNCs, we could look at this Italian experience of using autologous, fresh, and not manipulated PBMNCs in the treatment of diabetic patients with no-option CLTI as a strong indication of use arising from real-world data. Notably, ten randomized trials on G-CSF mobilized PBMNCs in patients with no-option CLTI demonstrated positive outcomes in terms of major amputation, AFS, wound healing rates, TcPO2 increases, and pain reduction, showing comparable results between naive and mobilized PBMNCs [79,80,162,163,164,165,166,167,168,169]. The cellular mobilization induced via G-CSF in regenerative therapy is a long-debated topic. Mobilization has been widely used; however, it has been suggested that the mobilization of CD34 + stem cells from the BM induced via the administration of G-CSF is not efficient in diabetic patients [138,140]. Accordingly, CD34+ mobilization did not appear useful in PB-MNC treatments; a study on patients with no-option CLI treated with pure CD34+ or non-mobilized PB-MNCs showed no differences in AFS [79,170]. Furthermore, another randomized trial, called the SCELTA TRIAL, suggests the “non-inferiority” of non-mobilized PB-MNCs compared to BM-MNCs [80].

In our metanalysis, all the centers that used this innovative immune-centric cell therapy were third-level reference centers with a high specialization in treating these patients. Furthermore, the substantial absence of both minor and major adverse effects confirms the high safety profile of this therapy [19,20,21,22,24,26]. Moreover, these data on PBMNCs’ safety were also reported in different meta-analyses on PAD, CLTI, and/or diabetic patients [17,171,172,173,174].

Point-of-care selective filtration for the concentration and immediate implants of naive PBMNC, in the same surgical procedure of wound debridement, reveals some specific advantages, such as minimal withdrawal invasiveness, which allows repeat cell implants several times. The clinical trials conducted thus far on CLI patients have employed varying cell implantation quantities and frequencies for cell therapy [14,17]. In a preliminary randomized controlled trial, patients who underwent four repeated injections of BM-MNCs showed an improvement in pain-free walking distance compared to those who received a single treatment [161]. Kang et al. further substantiated that multiple treatments were more efficacious than administering more cells in a single treatment [162]. Accordingly, Beugels et al. achieved the same result in angiogenesis using an immune-deficient rat model with hind limb ischemia [163]. All of these data are in line with the observation by Panunzi et al. [20], who observed that PBMNC cell therapy resulted in a steady increase in the TcPO2 levels that reached the maximum level after the second treatment, with a long period of maintenance for high-TcPO2 values after the third implant.

Moreover, Pall Celeris/MonoCells Solution System/HemaTrate Cook Regentec point-of-care (POC) selective filtration is a single-use device that is user-friendly and not operator-dependent. The procedure time to obtain PBMNC after a blood draw is no more than 12–15 min. The system is completely closed, so sterility is maintained.

From the clinical point of view, remarkably, this meta-analysis of six different studies [19,20,21,22,24,26] and 256 patients indicates that autologous fresh PBMNC implants are associated with a mean yearly amputation rate of 15.7%, which was the primary endpoint. Moreover, all included studies, except two [24,26], reported information on all-cause mortality, with a mean rate of 22.2% and a mortality rate of 18.8% at one year. One study comparing PBMNCs with the standard of care [19] reported a significant reduction in favor of PBMNCs (80% mortality in the control group vs. 20% mortality in the PBMNC group at two years).

Regarding the wound healing rate, a mean healing rate of 62%, with an average healing time of 208.6 ± 136.5 days was observed. One study comparing PBMNCs with the standard of care [19] reported a significant reduction in healing time in favor of PBMNCs.

As a soft endpoint, PBMNCs were also correlated with a TcPO2 increase, with an average increase of 22.7 mmHg and pain reduction in 73.5% of patients. No adverse events were reported for any patients.

Although the meta-analysis did not contain randomized studies, the rate of major amputation in the treated group was significantly lower than that in the data published in a previous study on patients with NO-CLI treated only with conventional therapy [9], showing a 15,7% amputation rate vs. 30%, respectively.

In addition, the meta-analysis showed a significantly reduced rate of major amputation compared to another study enrolling similar patients with BTA arterial disease and failed revascularization (15,7% vs. 36%) [6]. The significant reduction in the amputation rate, which correlated with the PBMNC implants reported through this metanalysis, is in line with what was observed in an extensive meta-analysis by Rigato et al. [17] of patients with no-option CLTI treated with autologous cell therapy: only PBMNCs and not BMMNCs or BMMSCs reduce the amputation rate and increase AFS.

Interestingly, the mortality rate was also significantly lower in this current meta-analysis than in the previously cited study: 18,8 vs. 50%, respectively, at one year [9]. Few diseases are associated with a higher death rate: only six of the 22 major forms of malignancy had a 5-year mortality rate higher than that of CLTI [12]. This incredibly high death rate highlights the need for novel treatment approaches to reduce major amputation in this vulnerable and precarious demographic. Scatena et al. [19] observed, in the control group, an 80% death rate at two years, while the PBMNC group’s mortality rate was reduced to 20% after a two-year follow-up. This result aligns with our meta-analysis’s mean mortality rate of 22.2%.

Our meta-analysis observed a mean healing rate of 62%, a promising result compared to the 26.1% healing rate in the group of diabetic patients with revascularization PAD reported in a recent study [175]. The same survey reported a 78.3% healing rate after successful revascularization [175]. Uccioli et al. [176], in a long-term comparison of 510 patients with diabetic foot ulcers who underwent revascularization and those who received no revascularization, reported a comparable healing rate in the revascularized group of 62.3% compared to the 62% obtained using PBMNC implants versus a no-option group healing rate of 48.1%. Interestingly, Ferraresi et al. reported a 44% wound healing rate for patients with no-option CLTI treated with hybrid foot vein arterialization at 10.8 ± 2 months of follow-up [177]. Accordingly, a recent meta-analysis of five observational studies comprising 208 patients (75% diabetic) with no no-option CLTI showed a complete wound healing rate of 53.4% [178]. A more extensive meta-analysis including 27 studies, 753 patients, and 793 limbs compared surgical and percutaneous foot-vein arterialization (FVA) outcomes at 6- and 12-month follow-ups [179]. The overall limb salvage rate at 6 and 12 months was 78.1% and 74.1% in the surgical FVA group, reaching 81.7% and 78.6% in the percutaneous FVA group, respectively. The wound healing rates reported only in the percutaneous FVA group were 64.5% at 12 months [179], comparable to the 62% observed in this meta-analysis.

Our meta-analysis regarding the secondary endpoint showed a baseline value of TcPO2 at 14.3 mmHg, indicating severe critical limb ischemia. A significative increase in TcPO2 compared to the baseline values was observed, with a mean value of 22.7 mmHg. Pain reduction was also observed, decreasing from a baseline value of 6.8/10, with a mean value of 1.8 points, and a percentage mean reduction in reported pain between the baseline and the endpoint of 73.5%.

These data, including oxygen tension evaluation and clinical information, suggest an improvement in foot blood perfusion, which allows for recovery from foot ischemia, as confirmed in post-treatment angiography. The TcPO2 and pain reduction results we observed overlap with those of many relevant studies that have described a significant increase in oxygen perfusion and a reduction in pain after PBMNC autologous cell therapy [77,159,174,180].

The International Union of Angiology’s Position Statement on No-Option CLTI recently included PBMNC cell therapy as the most promising cell therapy [23].

Global direct health spending on diabetes totaled around $700 billion in 2019, and it is projected to reach $825 billion by 2030; of this amount, medical expenses associated with diabetic foot ulcers (DFUs) will make up 33% of the overall costs associated with diabetes management [179]. Looking at economic sustainability Ragghianti et al. [21] evaluated the safety and cost-effectiveness of PBMNC injection in diabetic patients with chronic limb ischemia and small artery disease (SAD). This study aimed to assess the economic accessibility of this innovative PBMNC cell treatment system developed for use at the patient’s bedside or in the operating room. In this study, the beneficial effects on pain, TcPO2, and the avoidance of significant amputations in a considerable fraction of included patients—all patients allocated to major amputation—appear to be accessible compared to the costs incurred for other similar samples of patients with Texas grade 3 ischemic ulcers.

Authors [21] concluded that PBMNC therapy in diabetic patients with CLI and SAD and without CLI options appears to be beneficial in reducing the risk of major amputation. The median and mean total cost per patient were 8238 EUR ± 7798 EUR and 4426 EUR (3798 EUR; 8262 EUR), respectively.

Recently, nine studies establishing diabetic foot costs were included in a recent analysis of five European countries [181]. The total cost of amputation ranged from 15,046 USD in 2001 to 38,621 USD, including an estimated direct cost of amputation from 13,842 USD in 2001 to 83,728 USD during 2005–2009 and an indirect cost of amputation ranging from 1043 USD to 1442 USD. The total cost of an uninfected ulcer was 6174 USD in 2002, which increased to 14,441 USD in 2005. In summary, the cost of diabetic foot and its complications is exceptionally high, as diabetic foot costs more than major cardiac diseases [181].

*Study limitations:*

This meta-analysis shows several significant limitations that demand careful consideration. The meta-analysis was primarily based on monocentric observational studies, a factor that could limit the generalizability of the findings across diverse populations and clinical settings. Moreover, some included studies were single-arm observational studies without a control group, which hampers the ability to conduct comparative assessments of treatment efficacy. To overcome these challenges, future research must focus on conducting randomized clinical trials to provide more robust evidence. On a different note, as highlighted in the meta-analysis of Rigato et al. [17], “*the scientific community should interrogate on whether we still need additional evidence on this therapy, or we should recognize that cell therapy has the potential to modify the natural history of intractable CLTI. If this is the case, equipoise may not be granted in future RCTs*”.

Another limitation of this meta-analysis is correlated with different inclusion criteria, such as the location of ischemia (below the knee or the ankle) and infection status (infected or non-infected), which could introduce variability to the outcomes. Finally, the number of cell implant methods varied among the studies (two or three). It is also important to note that, despite the inherent variability of autologous cell therapy, all six studies included in the meta-analysis implanted PBMNCs harvested with the same point-of-care system based on selective filtration, starting with the same amount of blood drawn (120 mL) and obtaining the same amount of PBMNC cell concentrate in physiological saline (8–9 mL) with a superimposable total dose of implanted cells. Nevertheless, addressing these limitations in future research will be crucial, and it will have a significant impact on validating and extending the current findings, thereby advancing our understanding and treatment of ischemia.

This comprehensive discussion is crucial for the scientific community to grasp the results’ significance and potential implications fully.

## 5. Conclusions

From the above data, we may conclude that naive autologous PBMNC therapy is effective in reducing major amputation and mortality in diabetic patients with no-option CLTI. Increasing scientific evidence related to its action on vascular sprouting, the shift from M1 to M2 macrophages, and pain control, strongly supports its clinical use. The relatively easy method of concentrating mononuclear cells through a point-of-care device and the low cost of this therapy, together with the complete absence of side effects, also suggest its wider use in other, still-difficult clinical conditions to manage peripheral arterial disease in dialyzed patients or patients with chronic heart failure, conditions still characterized by high levels of major amputations and mortality [182].

PBMNC autologous cell therapy seems a feasible bail-out strategy for diabetic patients with NO-CLTI, considering that it is a safe and minimally invasive procedure. This treatment still represents an unmet medical need for these extremely critical patients. Due to the small number of studies and the lack of randomized trials, further research is needed. This systematic review and meta-analysis serve as a promising hypothesis-generating study.

## Figures and Tables

**Table 1:** PBMNC clinical studies included in the meta-analysis.

Authors	*n*	Study Design	Follow-Up (Weeks)	Age (Years)	Female %	Inclusion Criteria	Exclusion Criteria	Primary Endpoint (A); Secondary Endpoint (B)	Outcome	Ulcers’ Site/Median Area (cm^2^)
De Angelis et al. 2015 [26]	43	Nested case control	104	64.5 ± 17	41.9	Ischemic ulcers, Texas 1C,1D,2D,3D	Malignancy, coagulation disorders; systemic infection; HIV, hepatitis	A—limb salvage B—ulcer size, pain—VAS	Limb salvage: 95.3% PBMNC vs. 52.2% control *p* < 0.01	Not reported Ulcer size 80 cm^2^ (2–1200)
Persiani et al. 2018 [24]	50	Uncontrolled prospective	43	71.3 ± 12.6	32	NO-CLI DFU Texas 2–3	Malignancy, coagulation/hematological disorders, active infection	A—limb salvage, TcPo2 B—pain, VAS	Limb salvage 83.3% PBMNC. minor amputation rate 33.3% in PBMNC. TcPO2 from 16.2 ± 7.2 mmHg to 23.5 ± 8.4 mmHg (*p* < 0.001), VAS decreased from 9 ± 1.1 to 4.1 ± 3.3 (*p* < 0.001).	Not reported Ulcer size > 5 cm^2^
Scatena et al. 2021 [19]	38	Nested case control	104	77 ± 6.72	31.6	NO-CLI DFU Texas 2C-3C	Malignancy, lesions above the tibial–tarsal joint; moderate or severe infection NYHA class IV; anemia	A—amputation, mortality, healed patients B—TcPO2, healing time	89.5% PBMNC vs. 60.5% control (*p*= 0.0037)) Mortality at 2 y 20% PBMNC vs. 80% control group (*p* = 0.000). PBMNC 86.6% healed pts (33/38) vs. one patient healed in the control group (1/38) (*p* = 0.000)	PBMNC forefoot (78.9%); hindfoot (21.1%) Control—forefoot (73.7%); hindfoot (26.3%)
Panunzi et al. 2022 [20]	50	Uncontrolled prospective	78	75 ± 10	69	NO-CLI DFU Texas 3D	Malignancy, clinically active infection, life expectancy < 6 months, dialysis, ischemic lesions (immediate amputation)/extensive necrosis of the limb	A—TcPO2, major amputation, ulcer healing, back to walking, mortality	TcPO2 increase (17.2 ± 11.6 vs. 39.1 ± 21.8 mmHg, *p* < 0.0001), 84% limb salvage, ulcers healed with return to walking were observed in 60%, mortality 12%	Heel location 14% Dimension> 5 cm^2^ 50% of pts
Ragghianti et al. 2023 [21]	15	Uncontrolled prospective	52	69.8 ± 10	26.6	NO-CLI and SAD, DFU Texas 3C, 3D	Malignancy, coagulation disorders; systemic infection	A—composite endpoint of-TcPO2 at the first toe ≥30 mmHg and/or increase of at least 50% of TcPO2 and/or healing of the ulcer B—major amputation, healing rate, TcPO2	80% limb salvage, composite endpoint in 60.0% pts; t 6.7% healed within 90 days, 26.7% and 46.7% showed TcPO2 ≥ 30 mmHg and a TcPO2 increase of at least 50% at ninety days; 46.7% healed pts	Forefoot 12 (80.0%) Midfoot 1 (6.6%) Hindfoot 2 (13.3%) Median area cm^2^ 2.8 (0.7; 11.9)
Meloni et al. 2023 [22]	60	Uncontrolled prospective	52	74.8 ± 5.8	29.1	Unsuccess BTA revascularization, desert foot, TcPO2 < 30 mmHg.DFU Texas 1C,2C,3C, 1D,2D,3D	Malignancy, reduced life expectancy (less than 6 months), severe cognitive impairment	Major amputation, healing rate, survival TcPO2, pain (NRS)	69.1% healed and survived, 3.6% healed and deceased, 10.9% did not heal and deceased Limb salvage 83,6% (16.4% major amputation) TcPO2 values from 17 ± 11 to 41 ± 12 mmHg, (*p* < 0.0001) Pain values (NRS) from 6.8 ± 1.7 to 2.8 ± 1.7, (*p* < 0.0001)	

## Supplementary Materials

The following are available online at https://www.mdpi.com/article/10.3390/jcm13237230/s1: Figure S1: Study flow summary; Table S1: Detailed information on search string strategy.

## List of Abbreviations

ABI	Ankle brachial index
ACT	Autologous cell therapy
ADSCs	Adipose-derived stem cells
AFS	Amputation-free survival
BMMNCs	Bone marrow mononuclear cells
BMMSCs	Bone marrow mesenchymal cells
BTA	Below the ankle
CXCL1	Chemokine (C-X-C motif) ligand 1
CXCL5	Chemokine (C-X-C motif) ligand 5
CD34+	Hematopoietic stem cell positive to marker CD34
DFU	Diabetic foot ulcer
EPC	Endothelial progenitor cell
EVs	Extracellular vesicles
FAC	Fluorescence-activated cell sorting
FVA	Foot vein arterialization
G-CSF	Granulocyte colony-stimulating factor
HIF1α	Hypoxia inducible factor 1 subunit alpha
HSPCs	Hematopoietic stem/progenitor cells
IL-17A	Interleukin-17A
iNOS	Inducible nitric oxide [NO] synthase
INFγ	Interferon gamma
MCP-1	Monocyte chemoattractant protein 1
NF-kB	Nuclear factor-κB
NO-CLTI	No-option critical limb-threatening ischemia
PAD	Peripheral arterial disease
PBMNC	Peripheral blood mononuclear cell
POC	Point of care
RCT	Randomized controlled trial
ROS	Reactive oxygen species
SAD	Small artery disease
SAE	Serious adverse events
SPMs	Specialized pro-resolving mediators
SVF	Stromal vascular fractions
TcPO2	Transcutaneous oxygen pressure
Tregs	Regulatory T-cells
TNF-α	Tumor necrosis factor alfa
TNCs	Total nuclear cells
VEGF	Vascular endothelial growth factor
WFP	Wound-free period
WHT	Wound healing time RCT
