# Supplementary material for: The Immune-Centric Revolution Translated into Clinical Application: Peripheral Blood Mononuclear Cell (PBMNC) Therapy in Diabetic Patients with No-Option Critical Limb-Threatening Ischemia (NO-CLTI)—Rationale and Meta-Analysis of Observational Studies
Source: J Clin Med. 2024 Nov 28;13(23):7230. doi: 10.3390/jcm13237230 (PMC11642624; doi:10.3390/jcm13237230)
Supplement: Supplementary file 1 [file jcm-13-07230-s001.zip › jcm-3226439-supplementary.pdf]

SUPPLEMENTARY MATERIALS

Figure S1 – Study flow summary.

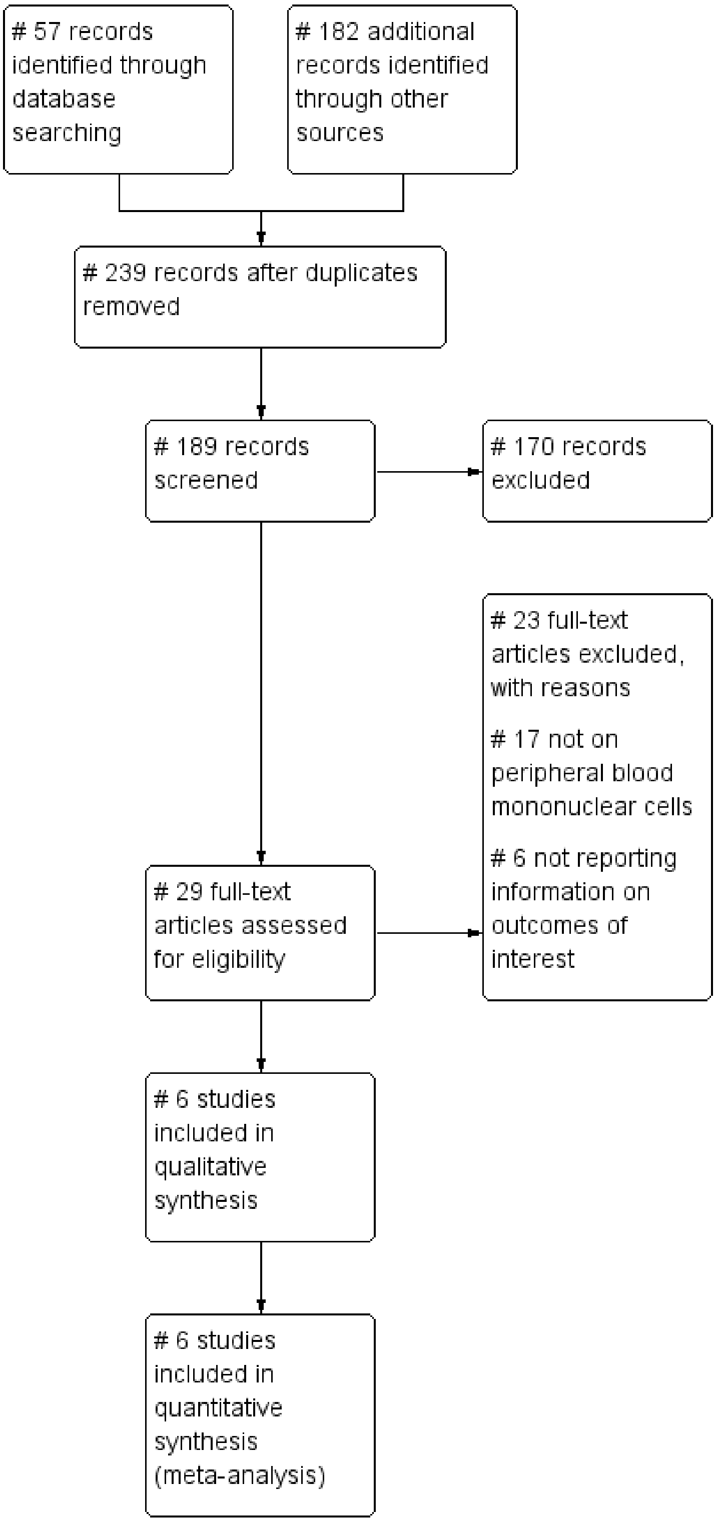

TABLE

Table S1 – Detailed information on search string strategy

**Limits:** any date up to Feb 1st, 2024
**MEDLINE** (N= 57 items)<br><br>**Search:** *(autologous OR "stem cells" OR "stem cell" OR "cell therapy" OR "cellular therapy") AND "ulcer" AND "diabetes" AND "peripheral".*<br><br>("autolog"[All Fields] OR "autologous"[All Fields] OR "autologic"[All Fields] OR "autological"[All Fields] OR "autologous"[All Fields] OR "autologously"[All Fields] OR "stem cells"[All Fields] OR "stem cell"[All Fields] OR "cell therapy"[All Fields] OR "cellular therapy"[All Fields]) AND "ulcer"[All Fields] AND "diabetes"[All Fields] AND "peripheral"[All Fields] AND "blood"[All Fields]<br><br>Translations<br><br>autologous: "autolog"[All Fields] OR "autologous"[All Fields] OR "autologic"[All Fields] OR "autological"[All Fields] OR "autologous"[All Fields] OR "autologously"[All Fields]<br><br>**EMBASE** (n=182 items)<br>(autologous OR 'stem cells'/exp OR 'stem cells' OR (('stem'/exp OR stem) AND ('cells'/exp OR cells)) OR 'stem cell'/exp OR 'stem cell' OR (('stem'/exp OR stem) AND ('cell'/exp OR cell)) OR 'cell therapy'/exp OR 'cell therapy' OR (('cell'/exp OR cell) AND ('therapy'/exp OR therapy)) OR 'cellular therapy'/exp OR 'cellular therapy' OR (cellular AND ('therapy'/exp OR therapy))) AND ('ulcer'/exp OR ulcer) AND ('diabetes'/exp OR diabetes) AND peripheral AND [embase]/lim NOT ([embase]/lim AND [medline]/lim) AND ('clinical trial'/de OR 'controlled study'/de OR 'randomized controlled trial'/de OR 'randomized controlled trial topic'/de OR 'retrospective study'/de)
**Additional search:**<br>Additional manual search of the references of included trials and former meta-analyses was carried out to identify other newly published and unpublished studies. Completed but yet unpublished studies with the procedures specified above were searched in the www.clinicaltrials.gov register. using the same search string as above.
